# Eliciting perspectives on remote healthcare delivery from service users with psychosis in the community: a cross-sectional survey study

**DOI:** 10.3389/fdgth.2024.1304456

**Published:** 2024-02-13

**Authors:** Ronja Kuhn, Nadia Abdel-Halim, Patrick Healey, Victoria Bird, Kathryn Elliot, Philip McNamee

**Affiliations:** ^1^ Unit for Social and Community Psychiatry, Centre for Psychiatry & Mental Health, Wolfson Institute for Population Health, Queen Mary University of London, London, United Kingdom; ^2^ Unit for Social and Community Psychiatry, East London NHS Foundation Trust, London, United Kingdom; ^3^ School of Electronic Engineering and Computer Science, Queen Mary University of London, London, United Kingdom

**Keywords:** remote healthcare, psychosis, technology, survey, digital exclusion

## Abstract

**Introduction:**

The transition towards remote healthcare has been rapidly accelerated in recent years due to a number of factors, including the COVID-19 pandemic, however, few studies have explored service users' views of remote mental healthcare, particularly in community mental health settings.

**Methods:**

As part of a larger study concerned with the development of a remotely delivered psychosocial intervention, a survey was conducted with service users with psychosis (*N* = 200) from six NHS trusts across England to gain cross-sectional data about service users' opinions and attitudes towards remote interventions and explore how digital access varies across different demographic groups and geographical localities.

**Results:**

The majority of service users had access to technological devices and a quiet space to receive care. Age was a key factor in motivation to engage with remote care as older participants had less access to technological devices and the internet, and reported less confidence to learn how to use new technologies compared to younger participants. Differences in access and attitudes towards remote care were found across the different geographical localities. Over half of the participants (53.1%) preferred a hybrid model (i.e., mixture of face-to-face and remotely delivered treatment), with only 4.5% preferring remote treatment exclusively. Factors that both encourage and deter service users from engaging with remote care were identified.

**Conclusions:**

The findings of this study provide important information about the environmental and clinical barriers that prevent, or limit, the uptake of remotely delivered care for people with psychotic disorders. Although service users often have the ability and capacity to receive remote care, providers need to be cognisant of factors which may exacerbate digital exclusion and negatively impact the therapeutic alliance.

## 1 Introduction

Advances in technology have precipitated an increasing shift towards the remote delivery of healthcare (via telephone or digital methods) over the past decade. The shift towards remote healthcare is in accordance with existing long-term plans set by key National Health Service (NHS) policies, e.g., The Five Year Forward View of Mental Health (1) and The NHS Long Term Plan (2), that proposed clear recommendations to further expand access to digital services for greater accessibility and choice. Given that around 21.3% of the population in England are living in rural areas (3) and have limited access to in-person treatment, one of the main benefits of the use of remote access to services is the removal of barriers to attendance and improved access for underserved populations. However, there are concerns that the increasing shift towards remote care may restrict therapeutic relationships between clinicians and service users, particularly within mental health provision (4), may cause risks to confidentiality and data security (5) and may lead to the digital exclusion of some population groups because of disparities in access due to demographic and socioeconomic factors. It is therefore of utmost importance to explore the demographic characteristics of specific populations to ensure that remotely delivered care does not perpetuate or exacerbate existing inequalities. A previous study (6) exploring factors impacting on uptake of remote therapy in a psychological therapy service for individuals with psychosis in London, identified older age, and socioeconomic status as key factors. Since London has the lowest rates of digital exclusion it was suggested that disparities in digital access may be more apparent in other regions in the UK, indicating the need to explore digital divide across the country.

Whilst many studies have examined advantages and disadvantages of remote mental healthcare, as well as its effectiveness, few studies have focused on mental health service users' views of remote care (5, 7). The closer involvement of service users in the development of digital care has been recommended by NHS England and evidence suggests that the inclusion of service users when designing remote interventions is linked to higher levels of adherence with digital healthcare (8). Since the successful implementation of remote mental healthcare is ultimately dependent on service users' levels of access to technology and their skills, as well as their confidence and motivation to engage with remote treatment, it is important to explore service users’ views and attitudes towards remote mental healthcare.

The increasing shift towards remotely delivered care, particularly as a response to the COVID-19 pandemic, means most service users have had at least some experience of receiving care in this way. This is therefore a useful time to make use of this opportunistic sample and explore service users' demographic characteristics and their views, and lived experience, of remotely delivered mental health care. For these reasons, this study used survey methods to engage with service users with psychosis from six NHS trusts covering different geographical localities in the UK to (a) gain insight into service users' opinions and attitudes towards remote interventions, (b) understand how digital access and technological skills vary across different demographic groups and geographical localities and (c) gain an understanding of service users' previous experiences with remote care. The study is nested within a broader research project concerned with the development of a remote care planning intervention for service users with psychosis, called Remote DIALOG+ (NIHR201680). Findings from the survey will be used to develop and improve the Remote DIALOG+ software, and limit digital exclusion, where possible.

## 2 Materials and methods

### 2.1 Design & setting

Responses to a survey questionnaire were collected between May and November 2022 to elicit perspectives on and experiences of remote mental health delivery from service users with psychosis in England. The survey was conducted in parallel with a focus group study (9), in order to provide a more quantitative understanding of the needs and attitudes of diverse demographic groups across geographic localities. The survey questions were co-produced with Patient and Public Involvement design panels consisting of service users and clinicians who were consulted during each stage of the project.

The study was given a favourable opinion by the North West-Preston NHS Research Ethics Committee in February 2022 (REC ref: 22/NW/0018**)**. All participant-facing study documents were reviewed by a panel of service users with lived experience prior to submission to the committee.

Service users participating in the survey were recruited from six NHS trusts: Cornwall Partnership NHS Foundation Trust, East London NHS Foundation Trust, Southern Health NHS Foundation Trust, Oxford Health NHS Foundation Trust, South West London & St George's Mental Health NHS Trust, and Sheffield Health and Social Care NHS Foundation Trust. This broad range of study sites was selected to ensure the inclusion of a broad range of demographics, including urban, rural and semi-rural environments and NHS Trusts were used as a proxy for urbanicity. Cornwall Partnership NHS Foundation Trust serves a wide and largely rural area. Staff travel many miles to visit service users at home and similarly, service users travel far distances to attend appointments at clinics. Similarly, Southern Health NHS Foundation Trust serves a largely rural and widespread area. On the other hand, East London NHS Foundation Trust and South West London & St George's Mental Health NHS Trust serve an urban area characterised by a high density of population and good access to services and transport links. Oxford Health NHS Foundation Trust and Sheffield Health and Social Care NHS Foundation Trust serve semi-rural areas that consist of cities, as well as suburban and rural areas.

The study relied on purposive sampling and clinicians working in community mental health teams (CMHT) and recovery centres across the participating NHS trusts were contacted in person, by telephone and by email and asked to identify potential participants from their caseload and inform them about the study and to assess eligibility to participate in the survey. With the permission of clinical teams, adverts were placed in CMHT facilities and recovery colleges to alert service users about the opportunity for involvement. Interested service users were able to reach out to researchers directly to learn more about the study.

### 2.2 Eligibility criteria

Service users were eligible for participation if they were above the age of 18, had mental capacity to consent, and a diagnosis of psychosis, defined as any F20–29 diagnosis within the International Classification of Diseases-10 (ICD-10) which includes schizophrenia, schizotypal, delusional, and other non-mood psychotic disorders. As psychotic disorders have high comorbidity with other mental health conditions, service users with multiple psychiatric conditions were deemed eligible to take part. All participants were actively receiving treatment from community care teams within the NHS trusts at the point of recruitment. A basic level of the English language was an additional inclusion criterion. Service users were not eligible for participation if they had no capacity to provide informed consent and were an inpatient in a psychiatric ward at the time of recruitment. Participants did not have to have any previous experience of remote care in order to be eligible.

### 2.3 Data collection

The survey was created to be completed online using the Queen Mary University of London Qualtrics XM platform, a cloud-based survey tool. Survey links were sent to eligible participants via several methods, but mostly via email. Participants were given the option to complete the survey alone or, if required, with the support of a researcher in-person in order to decrease sampling bias and to provide participants without access to the internet or technological devices the opportunity to participate in the survey.

Within the survey, all information captured was anonymous as no personal identifiable information was captured. All participants were assigned a unique participant ID number used for all data processing purposes. A consent form was embedded in the online survey. Participants had the right to withdraw from participating in the survey before submitting responses. The survey took approximately 10 min to complete and participants received a £5 voucher as compensation for their time.

### 2.4 Survey

The survey was divided into three sections. The first section was designed to ascertain participant characteristics, including age, gender, marital status, ethnicity, level of education, living situation, employment status, formal mental health diagnoses and the date of these diagnoses, and the initial treatment for these diagnoses. The second section consisted of six questions and was designed to collect data about access and attitudes to remote delivery of healthcare, e.g., “what technological devices do you have regular access to?” and “how confident do you feel in learning how to use new communication technologies?”. These questions ascertained practical elements of remote care usage, such as access to digital devices, access to a quite space and/or access to reliable transport. Finally, the third section consisted of eleven questions or statements (which were to be rated on a pre-determined scale) exploring previous experiences of remote treatment (telephone or digital) and treatment preferences e.g., “please rate how adequately remote treatment met your treatment needs?”. The questions in this section ascertained if, how and when participants had accessed remote care in the past, as well as treatment preferences for remote vs. face-to-face care. The final two questions were free text: “What factors, if any, would be most likely to deter you from accessing your treatment remotely?” and “What factors, if any, would be most likely to encourage you to access treatment remotely?” These open-ended questions were included to give respondents the opportunity to give any further, or more detailed views, that were not captured via the structured survey questions. The full survey is included in the Supplementary Materials.

### 2.5 Data analysis

All quantitative analyses were conducted using the Statistical Package for Social Sciences (SPSS) Version 27. Missing data was excluded from analysis. Levels of missing data were low across all questions (0.5%–2%) and missing data was equally distributed across the survey.

Data captured in the survey was analysed descriptively across all the outcomes related to demographics, attitudes, opinions and preferences for remote care. The two “free text” questions were analysed using conventional content analysis to look for repeated themes. A wide variation of data was collected and we were therefore not interested in quantifying it, but to directly derive coding categories from the data (10). Responses regarding factors that would encourage and deter service users from engaging with remote care were coded by two researchers, one with clinical experience (RK) and one with qualitative research experience (NH) and a set of categories were developed for each factor. These categories were discussed in multiple analysis meetings with a senior researcher (PM) with expertise in qualitative research.

## 3 Results

### 3.1 Demographics

Six surveys entries were excluded from the analysis due to being duplicates and four entries were excluded due to being clinicians and/or researchers completing the survey for testing purposes. A thorough process of data cleaning allowed any duplicate data or non-participant data to be identified and removed from the dataset. This resulted in a final sample of 200 service users completing the survey from the six study sites: East London NHS Foundation Trust (*n* = 36, 18%), Cornwall Partnership NHS Foundation Trust (*n* = 28, 14%), Oxford Health NHS Foundation Trust (*n* = 46, 23%), Southern Health NHS Foundation Trust (*n* = 34, 17%), South West London & St George's Mental Health NHS Trust (*n* = 29, 14.5%), and Sheffield Health and Social Care NHS Foundation Trust (*n* = 27, 13.5%).

Over half of participants were male (59%) and identified as White British (61%). 14% of the sample identified as Black/Black British and 8.5% identified as Asian/Asian British which was a representative sample of the general population of the UK (11). Close to half the sample (46%) had been diagnosed with a F20–29 diagnosis over five years ago and nearly half of the participants (45%) began treatment over five years ago. Over half of the population (55%) completed tertiary or further education and 31% completed secondary education. The majority of the participants (61%) were unemployed. Close to half of the sample (49.5%) were living with a partner, family or friends. Table 1 provides more detailed information of the demographic characteristics of the sample.

**Table 1:** Demographic information of the survey sample.

		*N* (%)
Age	18–24 years old	38 (19%)
	25–34 years old	47 (23.5%)
	35–44 years old	41 (20.5%)
	45–59 years old	61 (30.5%)
	60+ years old	13 (6.5%)
Gender	Woman	80 (40%)
	Man	118 (59%)
	Prefer not to say	2 (1%)
Education	Primary education or less	10 (5%)
	Secondary education	62 (31%)
	Tertiary/further education	110 (55%)
	Other general education	11 (5.5%)
	Not known	7 (3.5%)
Living situation	Living alone	79 (39.5%)
	Living with a partner or family	92 (46%)
	Living with friend(s)	7 (3.5%)
	Living in shared accommodation	21 (10.5%)
Employment	Employed full-time	39 (19.5%)
	Employed part-time	28 (14%)
	Student	11 (5.5%)
	Unemployed	122 (61%)
Ethnic group	White British	122 (61%)
	White Irish	1 (0.5%)
	White Other	11 (5.5%)
	Black/Black British-African	17 (8.5%)
	Black/Black British-Caribbean	11 (5.5%)
	Other Black/Black British background	3 (1.5%)
	Asian/Asian British-Indian	2 (1%)
	Asian/Asian British-Bangladeshi	3 (1.5%)
	Asian/Asian British-Pakistani	4 (2%)
	Other Asian/Asian British background	6 (3%)
	Mixed-White and Black African	1 (0.5%)
	Mixed-White and Black Caribbean	6 (3%)
	Mixed-White and Asian	3 (1.5%)
	Other mixed background	7 (3.5%)
	Chinese	2 (1%)
	Other ethnic group	1 (0.5%)

### 3.2 Access and attitudes to remote healthcare

#### 3.2.1 Access to technology, quiet space and transport

Overall, the majority of participants had access to a smartphone (84.8%), a reliable internet connection (85.2%), access to a quiet room (87.4%) and access to reliable transport to reach treatment sessions in-clinic (84.9%). Internet, smartphone and transport access decreased with age, whilst access to quiet space increased with age. Access to transport was the lowest among participants in Cornwall but access to quiet space was the highest, whereas access to quiet space was the lowest in East London. Internet and smartphone access was the lowest among participants from Sheffield and Southern Health. Table 2 shows the proportion of those who had access to a smartphone, internet, quiet space and transport across age groups and sites.

**Table 2:** Access to smartphone, internet, quiet space and transport across age groups and sites.

	Smartphone access	Internet access	Quiet space	Transport
Age group				
18–24	100%	94.6%	84.2%	92.1%
25–34	93.6%	93.6%	87.0%	85.1%
35–44	92.5%	87.2%	87.5%	87.5%
45–59	73.3%	75.4%	88.5%	82%
60+	38.5%	66.7%	92.3%	69.2%
Site				
Cornwall	92.9%	92.9%	96.4%	64.3%
East London	100%	83.3%	75%	88.9%
Oxford	88.9%	90.7%	93.3%	80%
Sheffield	63%	80.8%	85.2%	92.6%
SW London	93.1%	89.7%	86.2%	100%
Southern health	66.7%	73.5%	87.9%	85.3%

#### 3.2.2 Confidence using technology

**Figure 1:**
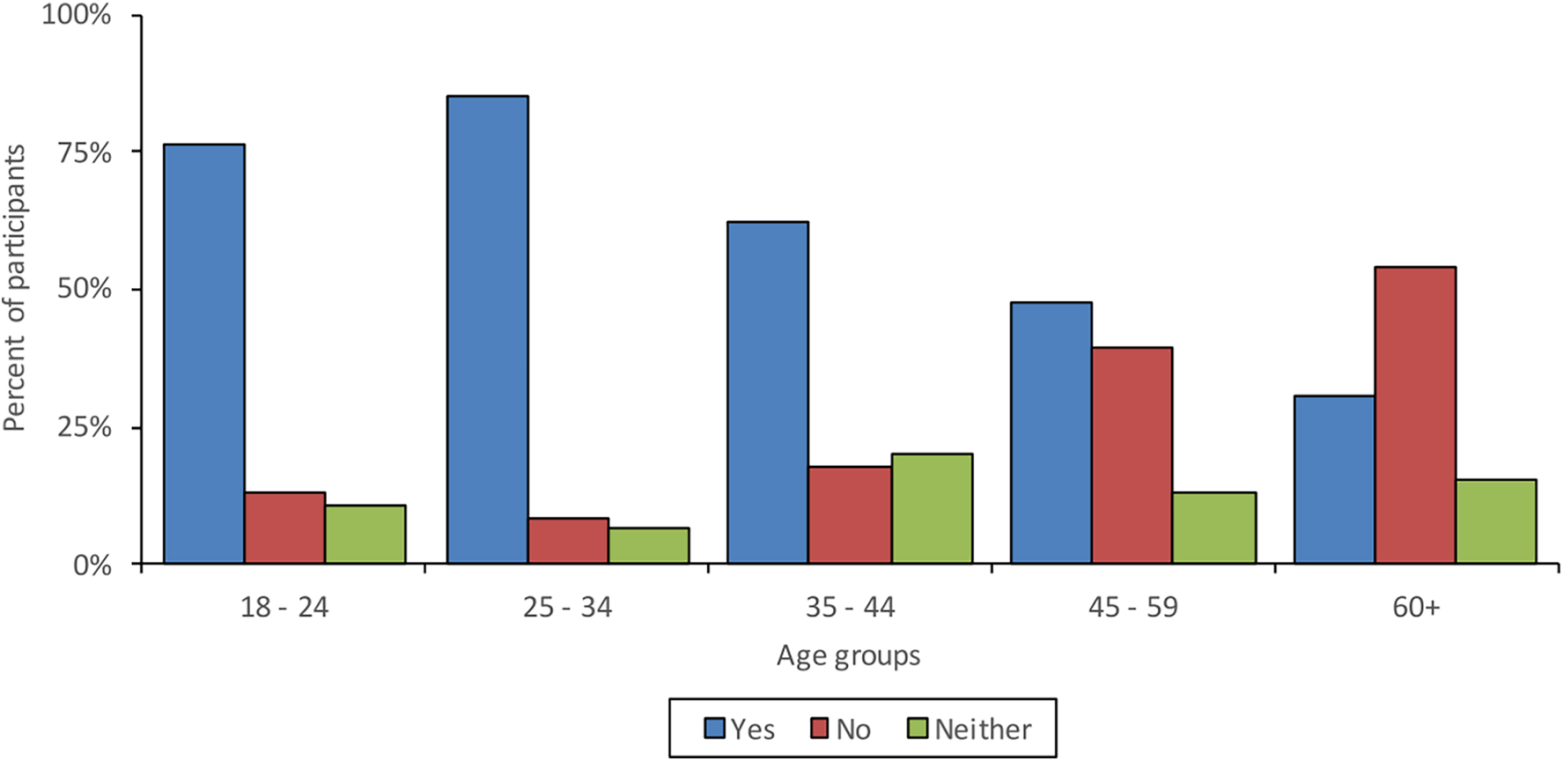
Confidence in learning how to use new communication technologies across age groups.

**Figure 2:**
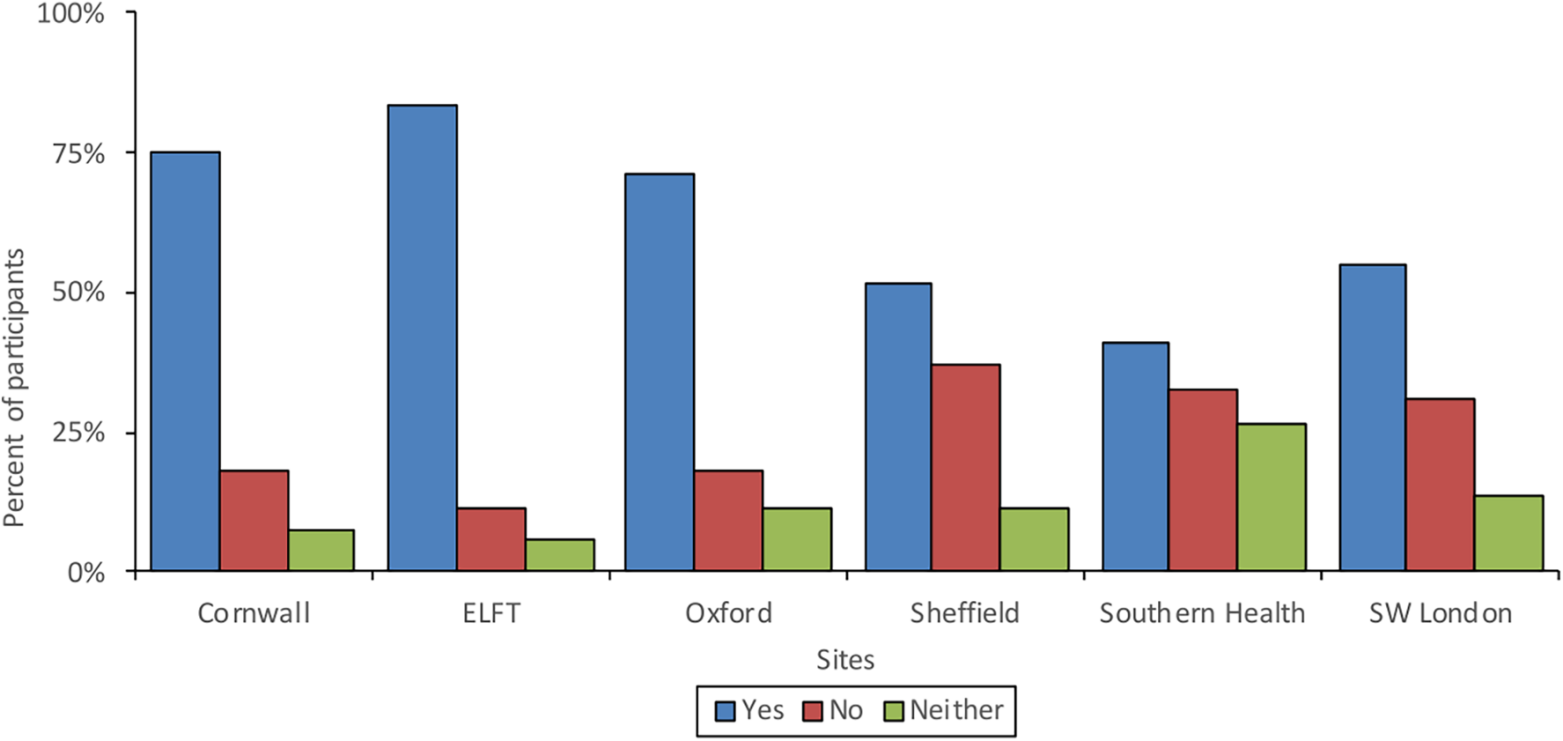
Confidence in learning how to use new communication technologies across sites.

63.8% of participants felt confident learning how to use new communication technologies. Confidence was found to decrease with age and participants in Southern Health and Sheffield were the least confident, whilst participants in East London were the most confident. Figure 1 shows the proportions of those who felt confident learning how to use new technologies across age groups and Figure 2 shows proportions of confidence across the different sites.

#### 3.2.3 Treatment preferences

If given the choice, 31.8% of participants indicated they would choose only face-to-face treatment where possible, whilst, 37.9% would prefer a mixture of face-to-face and remote treatment with most sessions being face-to-face, 4.5% would prefer only remote sessions, 15.2% would choose a mixture of face-to-face and remote treatment with most sessions being remote and 10.5% would not care if sessions are remote or face-to-face. Younger participants had a preference for hybrid treatment (a mixture of face-to-face and remote), whilst preference for face-to-face treatment exclusively increased with age. The majority of participants in East London and South West London would prefer hybrid treatment or did not have a preference, whereas in Sheffield more than half would prefer only face-to-face treatment. Table 3 shows treatment preferences across age groups and sites.

**Table 3:** Treatment preference across age groups and sites.

Treatment preference	Only face-to-face	Only remote	Mixture with mainly face-to-face	Mixture with mainly remote	No preference
Age group					
18–24	18.9%	0%	54.1%	21.6%	5.4%
25–34	19.1%	12.8%	38.3%	14.9%	14.9%
35–44	20%	0%	42.5%	17.5%	20%
45–59	52.5%	4.9%	24.6%	13.1%	4.9%
60+	53.8%	0%	38.5%	0%	7.7%
Site					
Cornwall	35.7%	10.7%	35.7%	10.7%	7.1%
East London	16.7%	10.7%	44.4%	16.7%	19.4%
Oxford	29.5%	2.3%	40.9%	18.2%	9.1%
Sheffield	51.9%	7.4%	25.9%	7.4%	7.4%
SW London	24.1%	3.4%	37.9%	24.1%	10.3%
Southern health	38.2%	2.9%	38.2%	11.8%	8.8%

#### 3.2.4 First session

Overall, 79.4% indicated they would prefer to have their first session with their clinician face-to-face. Older participants had a clear preference for their first session being face-to-face in comparison to younger participants, especially amongst the 25–34 year old group where only 62% said they would want their first session to be face-to-face, whereas every other age group had more than 80%.

### 3.3 Experiences of remote treatment delivery

#### 3.3.1 Previous experiences

**Figure 3:**
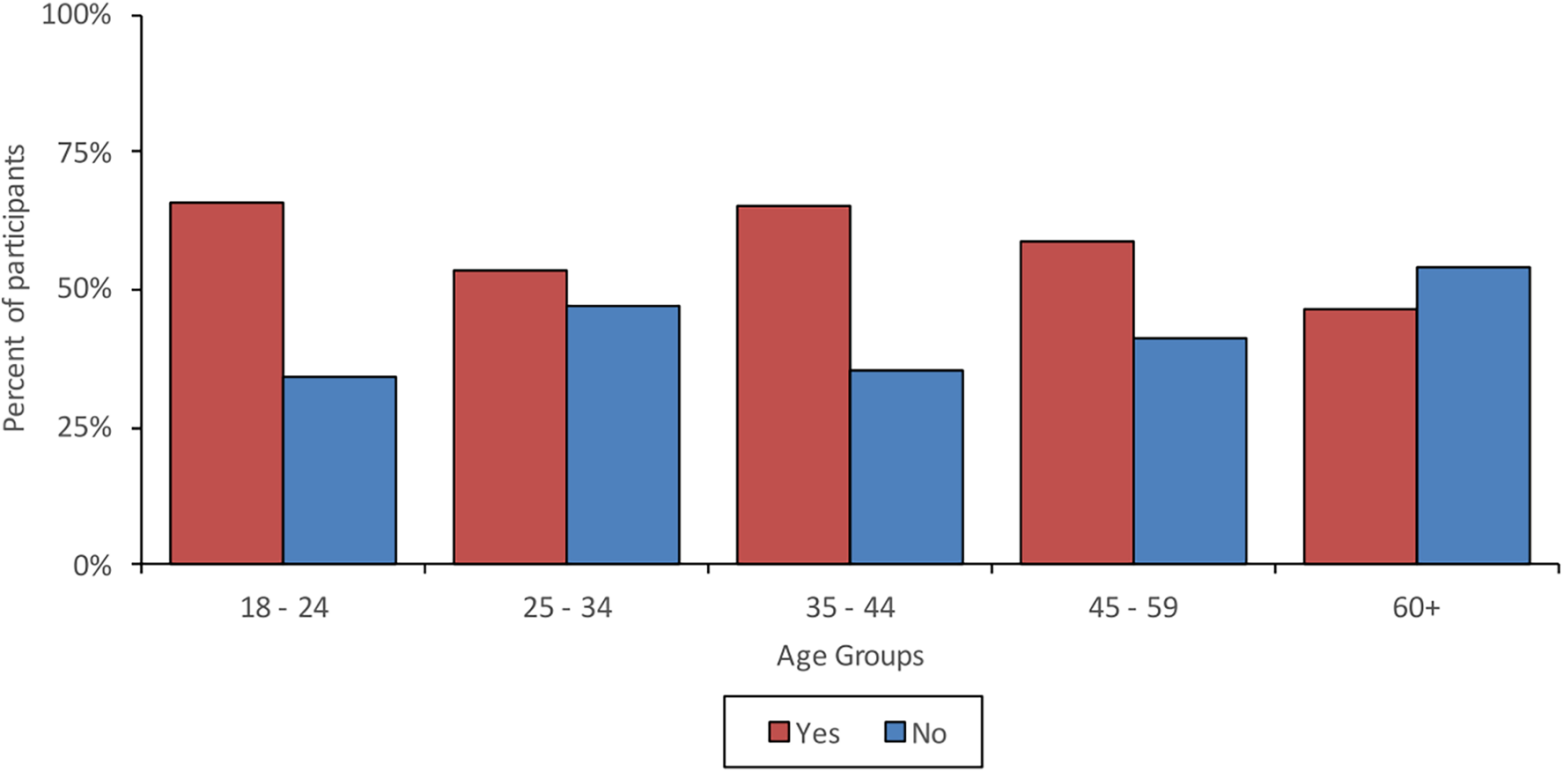
Previous remote care experiences across age groups.

**Figure 4:**
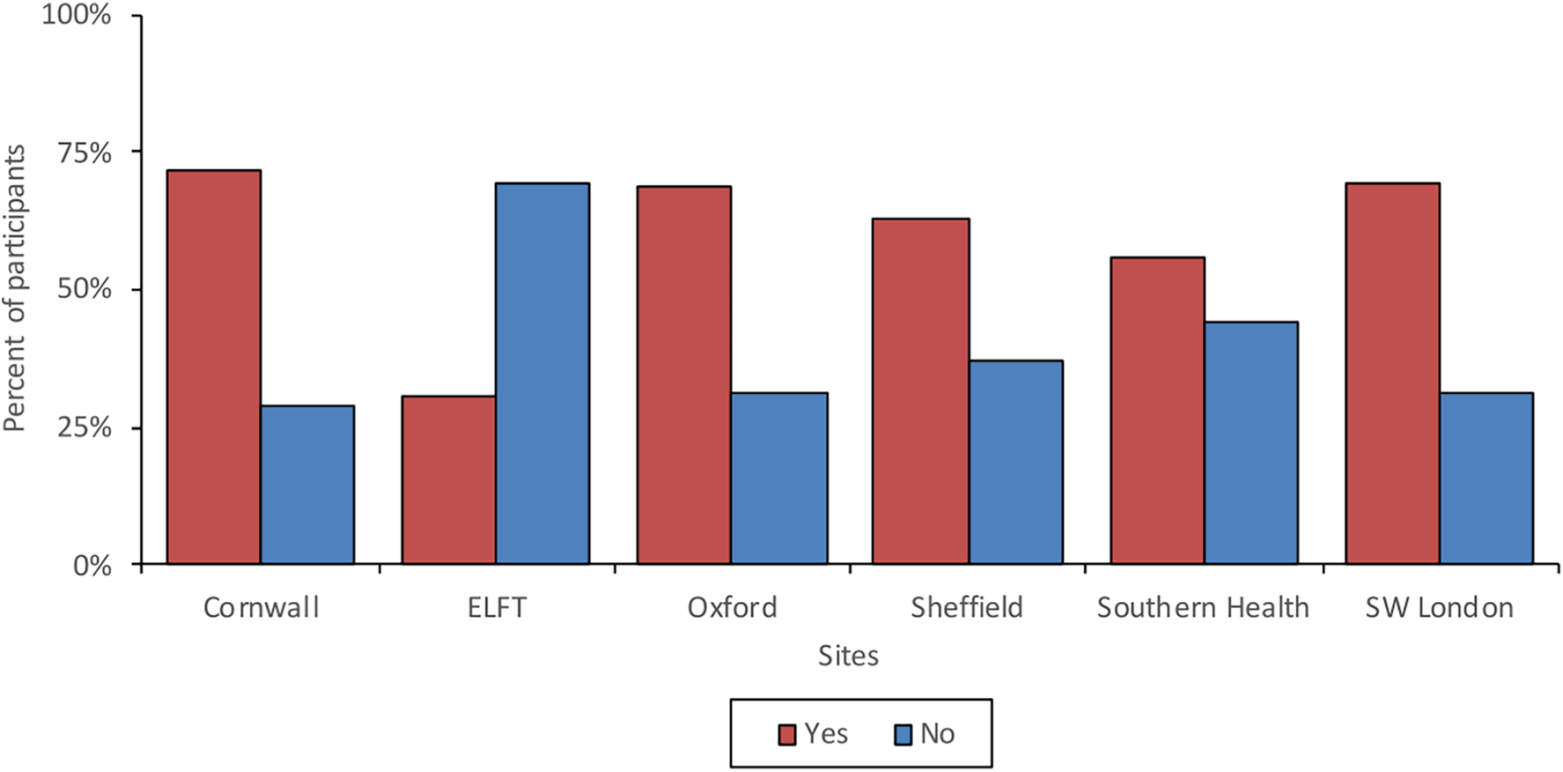
Previous remote care experiences across sites.

59.3% of the respondents had previous experience of receiving treatment that was not delivered face-to-face (meaning treatment was delivered over the phone or remotely using a web communication platform). Younger participants were more likely to have received remote treatment in the past compared to older participants. Of the participants that had received previous remote treatment, 69.2% indicated that this was due to the COVID-19 pandemic and related social distancing policies. Mobile phone/landline (40.1%) was the most used method for receiving remote treatment, followed by Microsoft Teams (27.9%) and Zoom (19.1%). 65.3% reported that previous remote treatment had met their needs. Younger participants reported higher satisfaction with remote treatment than older participants. Participants in East London were most satisfied with remote treatment, however they had the lowest rate of previous experience, and participants in Southern Health were least satisfied. Figure 3 shows the proportions of those who have received previous remote treatment across age groups and Figure 4 shows the proportion of those with experience of previous remote treatment across the survey sites.

### 3.4 Engaging with remote treatment—encouraging and deterring factors

Responses to the open-ended questions on the survey were analysed using conventional content analysis (10). Analysis led to two main themes (a) Encouraging factors and (b) Deterring factors. Each of these themes were comprised of associated categories which are listed and described below. Examples for each category are listed in Table 4.

**Table 4:** Example text extracts for each category for encouraging and deterring factors.

	Survey quotes
Encouraging factors—categories	
1. Physical and mental health conditions and vulnerabilities	“If I felt unwell and unable to attend this would be useful”
2. Home is comfortable and safe	“My home is more comfortable and NHS rooms were small with old furniture -therapy is uncomfortable, so the environment is important”
3. Improving scheduling flexibility for service-users	“It will allow more appointments and flexibility for both patients and staff, will be easier to access the appointment”.
4. Expanding patient choice and convenience	“Convenient, can attend without transport issues”.
5. Access to digital devices and support	“Technical help and support to set it up”
6. Improving access in time of crisis	“An accessible option for emergency meetings or to be clearly pointed in the right direction to other resources”.
Deterring factors—categories	
1. Affordability and accessibility of remote care	“I have no idea about computers and no reason to learn, I don't have internet at home”
2. Hampered communication, body language and therapeutic relationship	“No personal connection, hard to read facial expressions or body language”
3. Digital paranoia and distress	“Feel more anxious on the phone. It feels like a game or simulation on the phone”.
4. Confidentiality and privacy risks online	“It not being confidential, someone listening in”
5. Social withdrawal and isolation	“Sometimes when I’m ill I find it difficult to reach out which means I would be ill on my own. Yet if I had an actual face to face appointment I would try and be there”.
6. Perceived lower standards of care	“Feeling like it wasn't very effective/worth doing”

#### 3.4.1 Encouraging factors

Coding resulted in the following six categories associated with encouraging uptake of remote mental health care; (1) physical and mental health conditions and vulnerabilities, (2) home is comfortable and safe, (3) improving scheduling flexibility for service-users, (4) expanding patient choice and convenience, (5) access to digital devices and support, and (6) improving access in time of crisis. Table 4 shows example data extracts for each category.

##### 3.4.1.1 Physical and mental health conditions and vulnerabilities

Service users noted that remote appointments are a good treatment option when not physically well enough to travel to services due to physical health or mobility issues. Others reported not wanting to leave the house or meet people face-to-face due to anxiety, depression and agoraphobia and they expressed that it is “*easier to go through the motions*” when having a remote appointment. One service user reported preferring remote care due to fear of COVID.

##### 3.4.1.2 Home is comfortable and safe

Some service users reported preferring to attend appointments from the comfort of their own home and they described their homes to be safe, private and comfortable spaces. Others reported preferring to stay home and not having to leave their house due to weather-related issues, feeling more comfortable at home and going outside being fatiguing for them.

##### 3.4.1.3 Improving scheduling flexibility for service-users

Service users reported liking the flexibility of scheduling remote appointments. They reported remote appointments fit better around their work schedules and busy lives. They reported being more likely to receive a remote appointment in comparison to an in-clinic appointment and being more likely to be able to choose the time of their appointment.

##### 3.4.1.4 Expanding patient choice and convenience

Service users noted that remote care is often convenient as it reduces travel time and cost, causes less interruptions to the day, and no transport has to be organised. Others reported remote care can shorten the length of sessions as they noted that face-to-face sessions can be lengthy and time-consuming. Some reported wanting to be able to access remote care when travelling abroad for holidays or work and one service-user reported that remote care would be convenient if they moved away from the area. Others reported that remote care is convenient as it allows them to take notes on a computer during the session and access their records. Service users expressed that remote care might give them the opportunity to access a wider number of professionals and have group sessions with others. And some reported that clinicians are more likely to listen during remote sessions and provide more information, in comparison to face-to-face sessions.

##### 3.4.1.5 Access to digital devices and support

Service users reported that in order to be encouraged to engage with remote care, they would need to receive support on how to use and set-up technical devices. Furthermore, they reported needing access to devices and the internet. Some service users spoke about their previous positive experiences they had with remote care and identified this as an encouraging factor. Others reported being more likely to engage with remote care if they have an established (face to face) rapport with the clinician first.

##### 3.4.1.6 Improving access in time of crisis

Service users reported remote care being a good option when needing urgent help or when being in crisis. Some service users expressed they would engage with remote care if this would prevent them from being hospitalised, they needed urgent treatment or needed to be signposted to other services. Some reported that they would engage with remote care if in need and as a last resort when no other options are available.

#### 3.4.2 Deterring factors

The following six categories were developed when analysing factors that deter service users from engaging with remote care; (1) affordability and accessibility of remote care, (2) hampered communication, body language and therapeutic relationship, (3) digital paranoia and distress, (4) confidentiality and privacy risks online, (5) social withdrawal and isolation and (6) perceived lower standards of care. Table 4 shows example extracts for each category.

##### 3.4.2.1 Affordability and accessibility of remote care

Service users expressed feeling reluctant to engage with remote care due to a lack of digital literacy and not feeling confident using technology. They reported not having access to a quiet and confidential space, having unreliable or no internet access. Some service users reported having slow technical devices, not having access to any devices or not being able to afford any. Concerns about having problems with technology during sessions were expressed. Some participants expressed difficulties with scheduling remote appointments as they find them to be inflexible and only available at times when they are not available. Others reported not wanting to engage with remote care as they live close to services and have good transport links.

##### 3.4.2.2 Hampered communication, body language and therapeutic relationship

Respondents reported disliking remote treatment and preferring to see mental health professionals in-person as they find it easier to communicate and build a relationship face-to-face. Service users expressed that face-to-face treatment feels more comfortable, they enjoy the social aspect of going to the clinic and speaking to a clinician face-to-face can be “*grounding*”. Service users noted that they find it more difficult to express themselves during remote appointments due to language barriers and being unable to see each other's facial expressions and body language. Others reported finding remote care “*uncomfortable*”, “*boring*”, “*disconnected*” and “*isolating*”, disliking speaking to a screen and missing the human interaction they receive during in-clinic appointments. It was also reported that these communication difficulties during remote sessions can be exacerbated by auditory hallucinations they are experiencing.

##### 3.4.2.3 Digital paranoia and distress

Service users expressed that due to the nature of their illness they experience delusions and paranoia around technology. They reported feeling worried about their computer being hacked and others being able to listen to their confidential conversations and laughing at them. Service users reported that remote appointments can make them feel anxious and stressed.

##### 3.4.2.4 Confidentiality and privacy risks online

Service users expressed worries about engaging with remote care due to confidentiality issues. They reported feeling worried about others, such as family members or neighbours, listening to their sensitive conversations with mental health professionals. Some reported not having access to a safe space at home for confidential conversations. Service users reported needing reassurance that remote care is private and confidential.

##### 3.4.2.5 Social withdrawal and isolation

Service users reported finding it difficult to reach out to mental health professionals when feeling unwell. They noted that they would attend face-to-face appointments if feeling unwell but would not attend remote appointments and therefore isolate themselves further. Some service users reported finding it easier to lie about their mental health and hide symptoms of psychosis during remote appointments. They reported feeling worried about clinician's missing warning signs due to a lack of body language and non-verbal communication.

##### 3.4.2.6 Perceived lower standards of care

Service users expressed worries that remote care is not as effective as face-to-face treatment. They reported feeling that they are not getting checked properly and receive less information than during face-to-face appointments. As a result, they believed the quality of care may be lowered when remote healthcare is adopted.

## 4 Discussion

### 4.1 Overview

This survey study aimed to gain cross-sectional data about service users with psychosis' opinions and attitudes towards remote mental health care interventions, through drawing on their previous experiences with receiving remotely delivered community care and assessing their digital access and technological skills. The survey sample was recruited from six different NHS Trusts in England creating a representative sample of this service user group in terms of age, ethnicity, living situation and geographical setting.

### 4.2 Comparison with literature

The majority of service users with psychotic disorders did have access to a smartphone (84.8%), the internet (85.2%) and a quiet space (87.4%), indicating that logistically it is possible to deliver care remotely to a majority of this patient population. These figures are only slightly lower than those that have been reported in the general population where 94% of UK adults had internet access (12) and 82% had access to a smartphone (13). This is promising as previous studies have found those in contact with mental health services to be at risk of digital exclusion (14). In a meta-analysis (15), 66.4% of individuals with psychosis had a mobile phone and 49% had a smartphone, however these figures were expected to increase significantly over time, and the survey results here indicate this to be true. In a more recent study (6), figures were more in line with those reported in our study with 82% of service users with psychosis having access to a smartphone and 76% having internet access, indicating that access to technology has increased in the psychosis population over time. This proportion is only likely to further increase over time.

However, the results of our study also indicate that there is still a proportion of service users that do not have access to technology (15.2%), internet (14.8%) or a quiet space (12.6%), meaning that barriers to accessing remotely delivered mental healthcare still exist for a significant percentage of patients. In line with previous studies [e.g., (6)], age was found to be a key factor in access and motivation to engage with remote care. Internet and smartphone access decreased with age and older participants reported less confidence in learning how to use new technologies compared to younger participants (16). Previous studies have indicated that older adults are less confident in their abilities to use technological devices (17) and that excluded individuals find lack of knowledge to be a barrier to usage of technologies (18). In our study older participants also had less lived experience of receiving remote treatment (perhaps indicating a level of gatekeeping on behalf of clinical services), but those that had indicated less satisfaction with the remote treatment in comparison to younger participants. Furthermore, older participants having less confidence, as well as less experience, perhaps indicates a self-fulfilling loop whereby older people have less experience and therefore less confidence with such treatment. Interestingly, access to quiet space increased with age, but inversely, access to reliable transport decreased with age. Older participants having more access to quiet space and less access to reliable transport is reflective of older adults being more likely to be home owners (19) and in this survey, the majority of older people lived alone, whilst younger participants predominantly lived with a partner or family. Furthermore, older individuals also experience more difficulties accessing public transport (20) and reduce the amount they drive or stop driving (21).

Differences found amongst the age groups might also explain some of the variation found in access to technology, transport and quiet space across the different geographical localities. Service users in Cornwall had the most access to quiet space, but the least access to reliable transport, whilst participants in South West London and East London had the least access to quiet space but the most access to reliable transport. This is perhaps due to Cornwall's ageing population and being a wide-spread rural area with poor transport links, in comparison to London's younger, high-density population with good transport links but a smaller amount of home ownership. Differences were also found around previous remote care experiences and whilst three quarters of participants from Cornwall had previously received remote treatment, only a small proportion of participants from East London had, possibly due to closer clinics and better transport links. Again, a link between limited access to technology and low confidence was found amongst the participants from Sheffield and Southern Health who had the least access to technology and also felt the least confident learning to use new technologies.

These factors imply that remote care could be a useful alternative to face-to-face treatment for older individuals and individuals living in rural areas, however, support and adaptations may be needed to encourage engagement and usage. Older participants reported a strong preference for face-to-face treatment and treatment preferences were varied amongst individuals living in rural areas. Participants in East London, South West London and Oxford reported a preference for hybrid care and interestingly, in Cornwall and East London, two areas with very different levels of urbanicity and population composition, about 10% of participants indicated a preference for only remote care. This indicates that there may be a demand for remote care for varying reasons. Not many studies have explored service users' views towards remote care and one of the few studies that did focus on service users' views on digital health interventions found largely positive attitudes towards remote care amongst early psychosis service users, but emphasis was put on the importance of choice and the need for technology to complement, rather than replace in-person care (5). A preference for hybrid care was reported in our sample with over half of the sample (53.1%) reporting a preference for mixture of face-to-face and remote care, whilst less than a third (31.8%) reported a preference for face-to-face treatment exclusively, and a small, but not insignificant, proportion (4.5%) reported only wanting to receive remote treatment. Taking into consideration participants' wide-ranging views and attitudes towards remote care (evidenced by the often contradictory findings in the open text questions), it is crucial that a nuanced approach is adopted in how remote care is developed, offered and implemented. For remote care to be successful and meet service users' needs these wide-ranging views will need to be taken into account.

About 65% of participants that had previous experiences with remotely delivered care, reported that remote treatment met their needs, which although encouraging, also indicates the need to further explore service users' expectations and needs when accessing remote care in order to increase their satisfaction with it. Exploring how software, platforms and interfaces can be developed with service user needs in mind is paramount to ensure that they are user-friendly and digital exclusion is not exacerbated. This seems particularly important for older individuals who were more likely to be less satisfied with remote care comparative to younger participants. A majority of participants indicated that they had received remote treatment due to the COVID-19 pandemic, however, about 30% of participants indicated that they had received remote treatment due to other reasons, indicating that whilst the pandemic increased the need for remote care and was the majority of people's first taste of this form of treatment delivery, remote care was evidently accessed for other reasons, and reflects the increasing normalisation of remote care delivery within the NHS.

### 4.3 Strengths and limitations

There are several strengths of this study. Since access and attitudes towards remote care were assessed across different NHS trusts in England, the sample allows for a more in-depth picture of the digital divide across the country. Furthermore, the sample is representative of the demographic properties of the English population (in terms of age, gender and ethnicity) which allows for the results of the study to be abstracted to a larger population. Historically, minoritised ethnic groups have been under-represented in research, however, the sample included in this study is representative of the ethnic breakdown of the general population in England (11). The survey was conducted at a time when many people had used remote mental healthcare for the first time (due to COVID-19) and the survey made use of these lived experiences. In addition, the survey went beyond simply asking about device ownership and internet access, but used a more holistic approach to also enquire about environmental needs and barriers (such as availability of private spaces) and platform preference.

However, the study is also subject to limitations. As the survey was conducted online, it only included individuals who could access the internet, potentially leading to an overestimation of proportion of service users' access to technology as a whole and to sampling bias. However, all participants were offered the opportunity to complete the survey in collaboration with a researcher using NHS computers and therefore, personal access to the internet was not a requirement for participation. Secondly, since socioeconomic status has been linked to poorer digital literacy and digital divide (22), future studies should assess the role of sociodemographic status and multiple deprivation when exploring digital exclusion. In this study, NHS trusts were used as a proxy for level of urbanicity, and we do not have detailed information about the urban makeup of the different geographical locations. Collecting participant's individual postcodes as part of the study would have allowed the research team to ascertain scores on the Index of Multiple Deprivation (IMD) for each individual participant which could then be correlated with things such as wi-fi availability, digital device ownership and access to public transportation. As such the data collected was limited to descriptive analysis only, and the authors were limited in the amount of inferential statistical tests they could conduct. We recognise that collecting more forms of data could have strengthened the impact of this survey. Nonetheless, we believe that the descriptive statistics presented contribute an important snapshot of the views and attitudes of service users with psychosis in relation to digital literacy and remote healthcare delivery.

### 4.4 Implications

Taken together, these findings indicate that the majority of people with psychosis do have access to the technology needed to engage with remote mental health care. The numbers of internet non-users is ever declining (18) and the “digital divide” may be narrowing but some divide between older and younger individuals appears to remain. Older individuals appear to not only have less access to technological devices but also lack the confidence to use new technologies and have a clear preference for face-to-face treatment. As older individuals already experience social isolation due to geographic and transportation constraints, it is of importance that they are not further excluded from treatment provision. Access to adequate devices, as well as access to support and potential training around technology, is needed to increase confidence, ability and motivation to engage with remote care.

Various treatment preferences were reported and many service-users indicated preferring a hybrid model of care. Such hybrid models may be particularly beneficial for individuals with physical and mental health conditions that make it difficult to attend in-person clinic appointments, as well as individuals with busy schedules (work, childcare etc.) and individuals that are in crisis and need urgent support. Interestingly, in places where there were logistical difficulties and remote care would seem a good solution to address these, this did not always coincide with a general preference for remote care. For example, service users in Cornwall often have to travel long distances to clinics, indicating that remote care could increase access and decrease travel time. However, in this study participants from Cornwall did not report a preference for remote care. These findings suggest that a flexible, individualised, “no size fits all” approach is needed when providing service users with treatment options. It is of importance that a choice of face-to-face, remote and combination of both is given to service users in accordance to service users’ needs, abilities and circumstances rather than moving towards remote care as default, in order to increase access for underserved population groups. Furthermore, remote care cannot just be an attempt at replicating face-to-face care but rather advances in technology should be used to improve processes around the dynamism and flexibility of appointment scheduling and check-ups.

### 4.5 Conclusion

Until now, few studies have considered mental health service users' views of remote care. This study provides a comprehensive insight into the views of remote care held by people with psychotic disorders in England. Whilst the majority of participants had adequate access to technology, a significant proportion remain excluded due to digital poverty, particularly in rural areas. Age appears to be an important factor when assessing the accessibility and motivation to engage with remote care and whilst younger participants had more access to technology, felt more confident to use technology and prefer a combination of remote and in-person treatment, older participants continue to have poorer access to technology and prefer face-to-face treatment. Access to technology, the internet and quiet spaces, as well as previous remote care experiences and confidence using technology appear to vary across different locations in England, and have varying impacts on people's motivation to engage with remote care. Different factors that would encourage and deter service users from engaging with remote care were identified and many service users expressed a preference for hybrid treatment, indicating the importance of a flexible, individualised approach to treatment delivery. These findings provide important first steps in ensuring that future software is designed with end users in mind, and that practical guidelines and recommendations are created to limit digital exclusion of older adults with psychosis.

## Data Availability

The raw data supporting the conclusions of this article will be made available by the authors, without undue reservation.
