# Supplementary material for: Eliciting perspectives on remote healthcare delivery from service users with psychosis in the community: a cross-sectional survey study
Source: Front Digit Health. 2024 Feb 13;6:1304456. doi: 10.3389/fdgth.2024.1304456 (PMC10897019; doi:10.3389/fdgth.2024.1304456)
Supplement: Supplementary file 1 [file Table1.docx]

Supplementary Material

Eliciting perspectives on remote healthcare delivery from service users with psychosis in the community: A cross-sectional survey study

**Ronja Kuhn, Nadia Abdel-Halim, Patrick Healey, Victoria Bird, Kathryn Elliot, Philip McNamee***

*** Correspondence:** Philip McNamee: [philip.mcnamee@nhs.net](mailto:philip.mcnamee@nhs.net)

# Supplementary Data

**Survey**

**Demographic Questions**

Q1 Age Range

- 18 - 24 (1)
- 25 - 34 (2)
- 35 - 44 (3)
- 45 - 59 (4)
- 60+ (5)

Q2 Gender

- Woman (1)
- Man (2)
- Non-binary (3)
- Prefer not to say (4)
- Prefer to self-describe (5) __________________________________________________

Q3 Highest completed level of education

- Primary education or less (1)
- Secondary education (2)
- Tertiary/further education (3)
- Not known (4)
- Other general education (5) __________________________________________________

Q4 What is your living situation?

- Living alone (1)
- Living with a partner or family (2)
- Living with friend(s) (3)
- Living in shared accommodation (4)

Q5 What is your employment status?

- Employed full-time (1)
- Employed part-time (2)
- Student (3)
- Unemployed (4)

Q6 Main psychiatric diagnosis

________________________________________________________________

Q7 Other psychiatric diagnoses 
 (*please list all)*

________________________________________________________________

Q8 Ethnic group

- White British (1)
- White Irish (2)
- White Other (3)
- Black/Black British - African (4)
- Black/Black British - Caribbean (5)
- Other Black/Black British background (6)
- Asian/Asian British - Indian (7)
- Asian/Asian British - Bangladeshi (8)
- Asian/Asian British - Pakistani (9)
- Other Asian/Asian British background (10)
- Mixed - White and Black African (11)
- Mixed - White and Black Caribbean (12)
- Mixed - White and Asian (13)
- Other Mixed background (14)
- Chinese (15)
- Other ethnic group (16) __________________________________________________

**Access and Attitudes to Remote Delivery**

Q9 When did you first receive your psychiatric diagnosis?

- Within a year (1)
- Within the past 5 years (2)
- Over than 5 years ago (3)

Q10 When did you first begin treatment for this diagnosis?

- Within 6 months (1)
- Within a year (2)
- Within the past 5 years (3)
- Other than 5 years ago (4)

Q1 What technological devices do you have regular access to in your home?

 *(please tick all that apply)*

- Smart phone (1)
- Mobile phone that is not a smart phone (2)
- Laptop without camera (3)
- Desktop (4)
- Headset (microphone & headphones) (5)
- Earphones (with microphone built in) (6)
- Earphones (without microphone built in) (7)
- Tablet computer (e.g. iPad) (8)
- Landline (9)

Q2 Please respond to the following statement:

|  | Strongly disagre (1) | Disagree (2) | Neither/Nor Agree (3) | Agree (4) | Strongly Agree (5) |
| --- | --- | --- | --- | --- | --- |
| I feel confident learning how to use new communication technologies such as Zoom (1) |  |  |  |  |  |

Q3 Do you have a quiet room that you would feel comfortable speaking to your clinician freely in?

- Yes (1)
- No (2)

Q4 Do you have a reliable means of transport to reach your treatment sessions in-clinic?

- Yes (1)
- No (2)

Q5 Please rate

|  | Strongly disagre (1) | Disagree (2) | Neither/Nor Agree (3) | Agree (4) | Strongly Agree (5) |
| --- | --- | --- | --- | --- | --- |
| How much you enjoy the in-clinic element of treatment (1) |  |  |  |  |  |

Q6 Would you prefer to have your first session with your clinician online or face-to-face?

- Online (1)
- Face to face (2)

**Experiences of Remote Treatment Delivery**

Q1 Have you ever received treatment that was not face to face?

- Yes (1)
- No (2)

Q1a If yes, was this due to the COVID-19 pandemic and social distancing policies?

- Yes (1)
- No (2)

Q1b Please indicate what platform was used:

- Zoom (1)
- Microsoft Teams (2)
- Skype (3)
- Using mobile phone or landline (4)
- Other/ Not sure (5)

Q1c Please rate how much you agree with the following statement

|  | Strongly disagree (1) | Disagree (2) | Neither/Nor Agree (3) | Agree (4) | Strongly agree (5) |
| --- | --- | --- | --- | --- | --- |
| Remote treatment adequately met my treatment needs (1) |  |  |  |  |  |

Q2 If given the choice between remote treatment and face to face treatment, what would you choose?

- Only face to face treatment (1)
- A mixture of face to face and remote treatment with most sessions being face to face (2)
- I do not care if sessions are remote or face to face (3)
- A mixture of face to face and remote treatment with most sessions being remote (4)
- Only remote sessions (5)

Q3 Do you have a reliable internet connection in the space that you would access therapy from? 

Please note that this question is asking about your private space, not your home overall. 

- Yes (1)
- No (2)

Q4 Is there anyone that you would want to have with you in the room as you go through your routine treatment with your mental health clinician?

- Yes (1) __________________________________________________
- No (2) __________________________________________________

Q4a If yes, what is their relation to you (i.e. parent, sibling, carer, case worker, etc.)?

________________________________________________________________

Q5 Please respond to the following statement: I would prefer if my treatment was offered...

- Only face to face (1)
- A mixture of face to face and remote treatment with most sessions being face to face (2)
- I do not care if sessions are remote or face to face (3)
- A mixture of face to face and remote treatment with most sessions being remote (4)
- Only remote sessions (5)

Q6 What factors, if any, would be most likely to deter you from accessing your treatment remotely?

________________________________________________________________

Q7 What factors, if any, would be most likely to encourage you to access treatment remotely?

________________________________________________________________
